# A module-based analytical strategy to identify novel disease-associated genes shows an inhibitory role for interleukin 7 Receptor in allergic inflammation

**DOI:** 10.1186/1752-0509-3-19

**Published:** 2009-02-12

**Authors:** Reza Mobini, Bengt A Andersson, Jonas Erjefält, Mirjana Hahn-Zoric, Michael A Langston, Andy D Perkins, Lars Olaf Cardell, Mikael Benson

**Affiliations:** 1 Unit for Clinical Systems Biology, Department of Pediatrics, University of Gothenburg, Gothenburg, Sweden; 2 Department of Immunology, Sahlgrenska Academy, Gothenburg, Sweden; 3 Department of Experimental Medicine, Lund University, Lund, Sweden; 4 Department of Electrical Engineering and Computer Science, University of Tennessee, Knoxville, TN, USA; 5 Department of Oto-rhino-largyngeology, Karolinska Institute, Stockholm, Sweden

## Abstract

**Background:**

The identification of novel genes by high-throughput studies of complex diseases is complicated by the large number of potential genes. However, since disease-associated genes tend to interact, one solution is to arrange them in modules based on co-expression data and known gene interactions. The hypothesis of this study was that such a module could be a) found and validated in allergic disease and b) used to find and validate one ore more novel disease-associated genes.

**Results:**

To test these hypotheses integrated analysis of a large number of gene expression microarray experiments from different forms of allergy was performed. This led to the identification of an experimentally validated reference gene that was used to construct a module of co-expressed and interacting genes. This module was validated in an independent material, by replicating the expression changes in allergen-challenged CD4^+ ^cells. Moreover, the changes were reversed following treatment with corticosteroids. The module contained several novel disease-associated genes, of which the one with the highest number of interactions with known disease genes, *IL7R*, was selected for further validation. The expression levels of *IL7R *in allergen challenged CD4^+ ^cells decreased following challenge but increased after treatment. This suggested an inhibitory role, which was confirmed by functional studies.

**Conclusion:**

We propose that a module-based analytical strategy is generally applicable to find novel genes in complex diseases.

## Background

Most common diseases, including allergy and diabetes, are complex. The genetic susceptibility of an individual to such a disease appears not to be the result of a single causative gene but rather arises from multiple interacting genes. The identification of novel disease-associated genes is complicated not only by the large number of potentially relevant genes as well as the heterogeneity of the diseases. One approach to address this complexity is to arrange disease-associated genes in networks, where the genes form nodes and the interactions between the genes are represented by links between the nodes. Since disease genes tend to interact [1,2] the investigation may be facilitated by searching for sub-networks of co-expressed and interacting genes (such sub-networks will henceforth be referred to as modules). The rationale behind this is that in gene interaction networks, functionally related genes tend to form modules of tightly interacting genes [1,2]. Thus, such modules give an overview of the main disease mechanisms and could thus be dissected to find novel genes [3,4]. It is not known if such a module-based analysis is generally applicable to complex diseases that are caused by altered interactions between many different cell types and tissues. The hypothesis of this study was that such a module could be a) found and validated in allergic disease and b) used to find and validate one or more novel disease-associated genes (i.e. genes that either show disease-associated changes in expression or whose alleles influence the risk of disease). Allergy was chosen as a model of complex diseases because the main disease process is known and readily examined in both clinical and experimental studies; an allergen causes increased release of cytokines from type 2 T helper (Th2) cells which activate B cells, eosinophils and mast cells to release inflammatory proteins [5]. This disease process is thought to be common to different forms of allergy, such as seasonal allergic rhinitis (SAR), atopic eczema and asthma. It involves many well-documented genes that can be used as references to validate the bioinformatic analyses as well as to construct modules of co-expressed and interacting genes. Moreover, since disease genes tend to interact, interactions with known disease genes can be used as a criterion to select novel genes for functional validation. However, several observations indicate more complex disease mechanisms than implied by the Th2 induced disease process. Many other cell types and tissues have been implicated and genomic high-throughout studies have shown the involvement of hundreds of genes. Several of these genes do not appear to overlap in different forms of allergy or to have roles that can be explained by the Th2 paradigm [6-11].

Based on the underlying principle that disease-associated genes tend to interact, we first identified a gene with a key regulatory role for the disease. This was used as a reference gene to construct a module and then dissected that to find the novel gene that had most interactions with known disease-associated genes. This was achieved by combining integrated analysis of gene expression microarray data with experimental studies.

We propose that these module-based analytical principles may be generally applicable to find novel genes in complex diseases.

## Results

**Figure 1:**
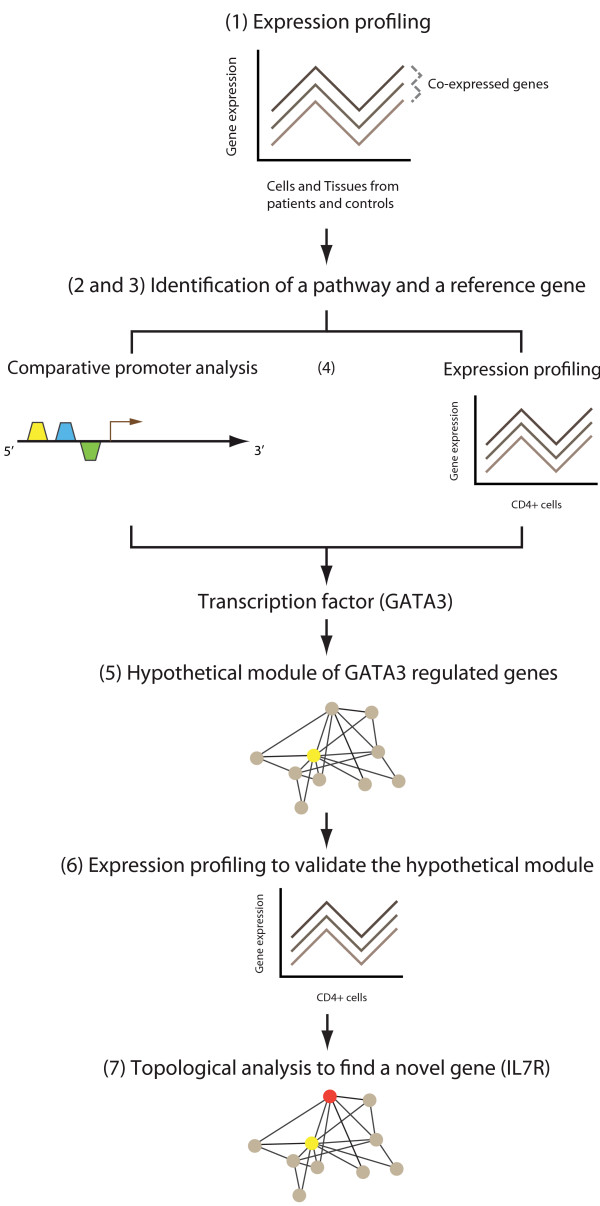
**An outline of the analytical process in the study**. 1) Integrated analysis of gene expression microarrays to find disease-associated genes, 2) Bioinformatic identification of a key pathway among those genes 3) Selection and experimental validation of a reference gene in the pathway, 4) Combined comparative promoter and gene expression microarray analysis to find a transcription factor that co-regulated the reference genes and other putative module genes, 5) Construction of a hypothetical module based on genes co-regulated by the transcription factor, 6) Independent gene expression microarray studies to validate the hypothetical module, 7) Topological analysis of module genes to find a novel gene. Experimental validation of the gene.

The hypothesis of this study has been that a module of disease-associated genes could be found and validated in allergic disease, and then used to find and validate one or more novel disease genes. A step-wise analytical approach was employed (figure 1):

First, a gene with key regulatory role for the disease was identified based on converging data from integrated analysis of gene expression microarray data from allergic patients and healthy controls, information in the literature and experimental validation studies. Next, this gene was used to build a hypothetical module of genes co-regulated by the same transcription factors (TFs). The underlying assumption was that functionally related genes, such as those in a pathway or a module, are often co-regulated by the same TFs. The TFs and their target genes were identified and organized into a module by combining comparative promoter analysis, gene expression data and known gene interactions. This hypothetical module was tested in an independent material, by examining if the expression changes of the module genes could be replicated. Finally, the module was searched for a novel disease gene that was validated by functional studies.

### 1) Identification of disease-associated genes by integrated analysis of gene expression microarray data from cells and tissues from patients with different forms of allergy and healthy controls

Combinatorial algorithms were used to extract groups of highly inter-connected genes (cliques) from graphs derived from transcriptomal correlation matrices containing millions of correlate pairs [12-14]. This resulted in the identification of 103 genes that will henceforth be referred to as disease-associated genes.

### 2) Identification of a common pathway among the disease-associated genes

A manually curated pathway database (Ingenuity Pathway Analysis, IPA) was used to identify pathways among the disease-associated genes (see additional figure 1). IPA is a bioinformatics program that is based on manual review of more than 200,000 scientific publications. The resulting network of direct physical, transcriptional, and enzymatic interactions between mammalian orthologues was used as a template to organize the disease-associated genes into statistically scored pathways. The pathway that received the highest statistical score was T cell receptor (TCR) signaling (p < 10^-6^), which included genes belonging to the TCR complex (*TCRA*, *TCRB*, *CD3Z *and *CD3D*) as well as down-stream genes, *ZAP70, LAT, LCK *and *ITK*. In addition, the remaining disease-associated genes were searched for genes that had direct interactions with those generated by the pathway database. Three other disease-associated genes fulfilled this criterion, namely *IKBKG *[15], *LEF1 *[16], and *MAP4K1 *[17]. The above mentioned genes will henceforth be referred to as TCR pathway genes.

### 3) Identification and experimental validation of a reference gene in the TCR pathway

**Figure 2:**
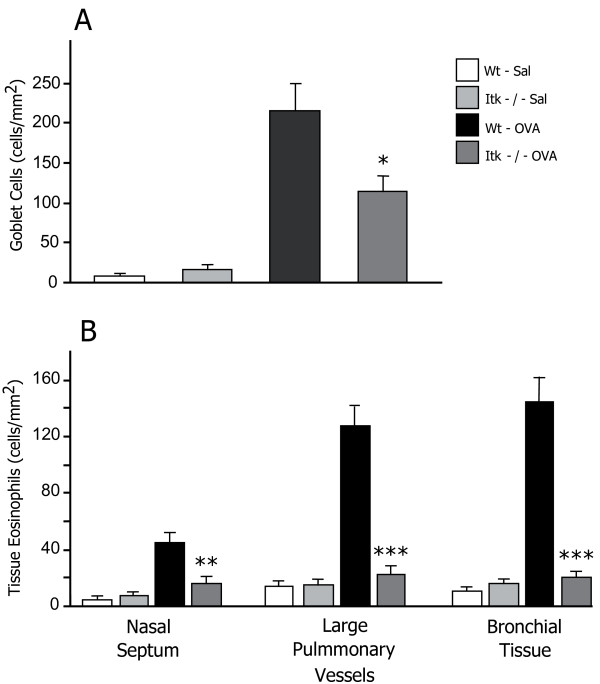
**The manifestation of an allergic inflammation is significantly reduced in mice lacking *ITK***. Numbers of PAS-positive bronchial goblet cells (A). EPO-histochemistry was used to quantify the tissue eosinophilia in the upper airways, bronchi, and around large pulmonary vessels (B). Data are presented as means ± SEM. Stars denote levels of statistical difference between OVA-treated wild-type and *ITK*-deficient animals (* = P < 0.05, ** = P < 0.01, *** = P < 0.001).

This step aimed to find a reference gene in the TCR pathway that in the next step would be used to build a hypothetical module of functionally related disease genes. We proceeded by examining the TCR pathway in allergen-challenged CD4^+ ^cells from patients with seasonal allergic rhinitis (SAR). Gene expression microarray analyses of these cells showed a more than a two-fold increase in expression of six of the TCR pathway genes; *CD3D*, *CD3Z*, *LCK*, *ITK*, *ZAP70 *and *MAP4K1*. Of these, *ITK *was selected as a putative reference gene because of its involvement in the polarization of Th2 cells and its location in a chromosomal susceptibility region for allergy, 5q31-33 [18]. The functional role of *ITK *was explored in an *in vivo *mouse model of allergic inflammation in the upper and lower airways. Allergen challenge resulted in a marked goblet cell hyperplasia in bronchi and bronchioles (figure 2A). This response was significantly reduced in similarly treated *ITK*^-/- ^animals. In both upper and lower airways the allergen exposure led to eosinophilia that was significantly reduced in *ITK*^-/- ^mice (figure 2B).

### 4) Identification of transcription factors that regulated *ITK*

**Figure 3:**
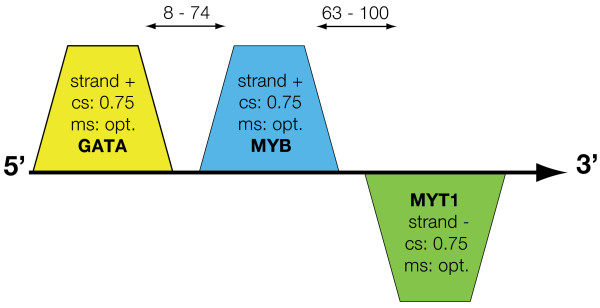
**Framework of three transcription factor binding sites**. The parameters and their abbreviations are: +, sense strand orientation; -, anti-sense strand orientation; cs, minimal core similarity; ms, minimal matrix similarity. Generally, cs was at default (0.75) and ms thresholds were the optimised default values for the respective matrices; distance, number of nucleotides to the next element (arrows in the upper part of the figure). *GATA*; strand, +; distance to next element, 8–74 bp. *MYBL*; strand, +; distance to next element, 63–100 bp. *MYT1*; strand, -.

Functionally related genes, such as those in a pathway or a module, are often co-regulated by the same transcription factors (TFs). Thus, if the TF that regulated *ITK *and other genes of the TCR pathway could be identified this information could be used to find other disease-associated genes and organize them into a hypothetical disease module. Comparative promoter analysis revealed a framework of TF binding sites that was shared by *ITK *and two other TCR pathway genes, *CD3D *and *LEF1 *(see additional file 1 for details). The framework contained TFBS for TF of the *GATA*, *MYB *and *MYT1 *families (figure 3). Gene expression microarray analysis showed that the corresponding TF, namely *GATA3, MYB *and *MYBL1 *increased in allergen-challenged CD4^+ ^cells (7-, 9- and 3-fold increases, respectively). To filter out the TF that was most relevant for Th2 polarization we analysed their correlations with *ITK *in gene expression microarray data from CD4^+ ^cells from 73 atopic patients and 19 non-atopic controls. The correlations between *ITK *to *GATA3, MYB *and *MYBL1 *were r = 0.68 (p < 0.0001), 0.41 (p < 0.001) and 0.25 (p < 0.01), respectively. Because *GATA3 *had the highest correlation coefficient it was chosen to construct a hypothetical module as described below.

### 5) Construction of a hypothetical module of genes co-regulated by *GATA3*

Since functionally related genes are often co-regulated by the same TF, we used *GATA3 *to construct a hypothetical module. We started by identifying 47 genes previously described to interact with *GATA3*. To filter out those of the interacting genes that were relevant for human CD4^+ ^cells we again analyzed the gene expression microarray data from the CD4^+ ^cells from the 73 atopic patients and 19 non-atopic controls. Genes whose expression values correlated with *GATA3 *(r > 0.4 and p < 0.0001) were considered as hypothetical module genes. This resulted in a module that comprised 37 genes, several of which had key roles in allergic disease, for example *IFNG *and *IL4R *(table 1).

**Table 1:** Genes included in the hypothetical module

**Gene symbol**	**Gene Name**
CCR4	Chemokine (C-C motif) receptor 4
CD28	Antigen CD28
CD3D	CD3 Antigen, delta subunit
CFLAR	CASP8- and fadd-like apoptosis regulator
CTLA4	Cytotoxic t lymphocyte-associated 4
EDG1	Endothelial differentiation gene 1
EED	Embryonic ectoderm development protein
ESR1	Estrogen receptor 1
GATA2	GATA-binding protein 2
GATA3	GATA binding protein 3
GPR44	G protein-coupled receptor 44
HIVEP2	HUMAN immunodeficiency virus type 1 enhancer-binding protein 2
HSPD1	Heat-shock 60-kd protein 1
IFNG	Interferon, gamma
IL4R	Interleukin 4 receptor
IL6	Interleukin 6
IL7	interleukin 7
IL7R	interleukin 7 receptor
IRF4	Interferon regulatory factor 4
IRS1	Insulin receptor substrate 1
ITK	IL2-inducible T-cell kinase
LEF1	Lymphoid enhancer-binding factor 1
LTA	Lymphotoxin-alpha
LTB	Lymphotoxin-beta
MAF	V-MAF avian musculoaponeurotic fibrosarcoma oncogene homolog
MAPK1	Mitogen-activated protein kinase 1
NFKB1	Nuclear factor kappa-b, subunit 1
NPPA	Natriuretic peptide precursor A
PP3CA	Protein Phosphatase 3 catalytic subunit, alpha isoform
PTCRA	Pre-t-cell receptor, alpha-chain precursor
PTPRJ	Protein-tyrosine phosphatase, receptor-type, J
RUNX3	Runt-related transcription factor 3
SH2D1A	SH2 domain protein 1A
STAT4	Signal transducer and activator of transcription 4
TCF12	Transcription factor 12
TCF3	Transcription factor 3
TNF	Tumor necrosis factor

### 6) Validation of the hypothetical module

**Figure 4:**
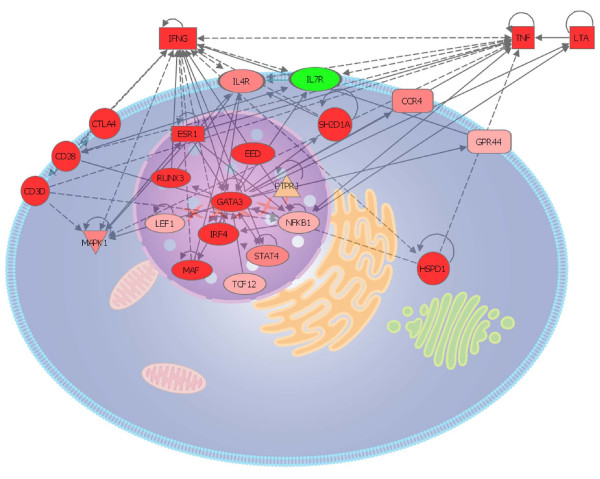
**Network representation of module formed by genes co-regulated by *GATA3 *in allergen-challenged CD4^+ ^cells from patients with seasonal allergic rhinitis**. The interaction network was generated using Ingenuity pathway analysis. Genes shaded in red were up-regulated whereas green were down-regulated. The color intensity reflects the significance value of the regulation. Solid and dotted lines represent direct and indirect interactions, respectively. Node shapes represent the class of molecule, for example donut shapes represent transcription factors (for detailed information see, )

To validate the module we hypothesized that the module genes would show significant gene expression changes in an independent material of allergen-challenged CD4^+ ^cells from 19 patients with SAR. Indeed, out of 33 genes that had detectable levels, 23 did change significantly. These genes formed a tightly connected module with an average of 3 ± 1 interactions/gene (figure 4). Since glucocorticoids are effective in treating allergic inflammation we hypothesized that this treatment would significantly reverse the expression levels of the 23 module genes. Indeed, 16 of the 23 genes satisfied this hypothesis.

### 7) Identification and validation of a novel disease gene in the module

**Figure 5:**
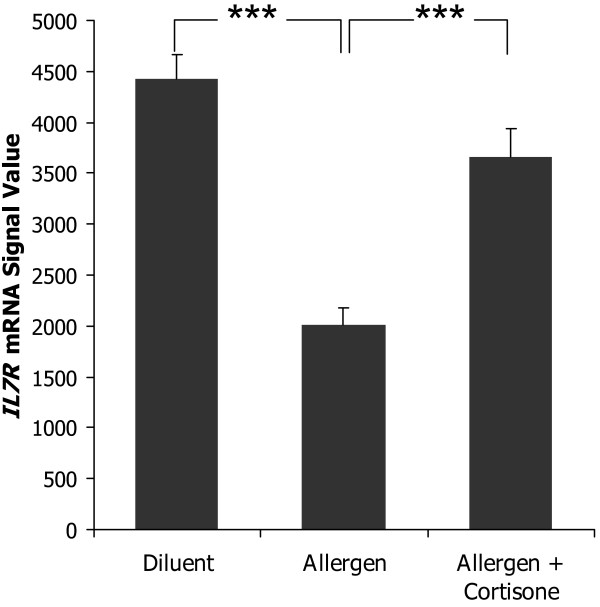
**Gene expression microarray analysis of *IL7R *expression in diluent-treated, and allergen-challenged CD4^+ ^cells with and without treatment with corticosteroids**. Data are presented as means ± SEM (*** = P < 0.001).

**Figure 6:**
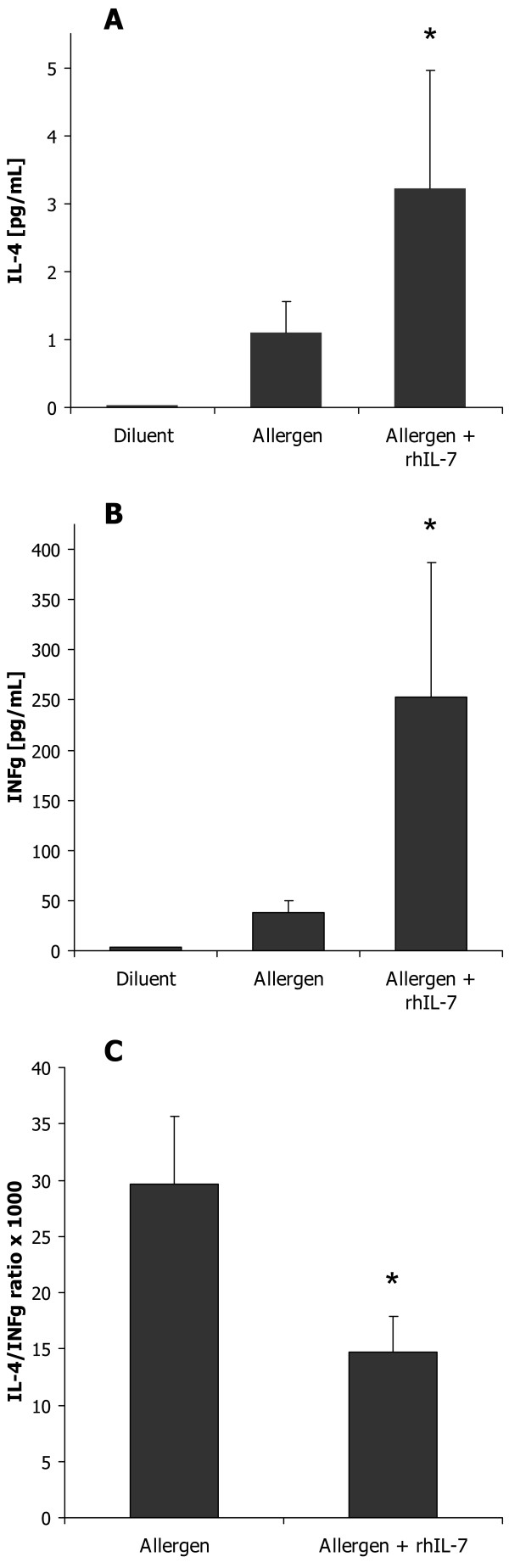
**A) IL-4, B) IFN-γ and C) the IL-4/IFN-γ^-3 ^in the supernatants of allergen-challenged PBMC from patients with seasonal allergic rhinitis, treated with diluent, or allergen with and without rhIL-7**. Data are presented as means ± SEM (* = P < 0.05).

Eleven of the 23 module genes that had been validated by the independent gene expression microarray study (figure 4) had not been previously been described in allergic diseases. These were *EED*, *ESR1*, *GRPR44*, *HSDP1*, *IL7R*, *LEF1*, *MAF*, *PTPRJ*, *RUNX3*, *TCF12*, and *SH2D1A*. Since disease-associated genes tend to interact [1,2] we computed the number of interactions between each of these eleven genes and the remaining, known disease-associated genes. The average number of interactions was 2.6 ± 0.4. *IL7R *had the highest number of interactions (n = 5) that included signature genes for Th2 activation like *IL4R *and *IFNG*. The expression levels of *IL7R *were lower in allergen-challenged CD4^+ ^cells compared to diluent-treated cells from patients, while treatment with corticosteroids increased the expression of *IL7R *(figure 5, both p < 0.001). Gene expression microarray analysis of an independent material of allergen-challenged CD4^+ ^cells from patients and healthy controls, also showed lower expression in patients, 3724 ± 512 vs. 5090 ± 519 (p = 0.003). These findings suggested the hypothesis that IL-7R signaling would inhibit the activation of Th2 cells. To test this hypothesis we examined if IL-7 would decrease production of the Th2 cytokine IL-4 from allergen-challenged PBMCs from allergic patients. However, IL-7 stimulation had the opposite effect; a 3-fold increased production of IL-4, 1.10 ± 0.45 compared to 3.22 ± 1.73 pg/mL, p < 0.05 (figure 6A). Similarly, the Th2 cytokines IL-5 and IL-13 also increased following IL-7 stimulation (see additional file 1). This suggested an alternative hypothesis, namely that IL-7R signaling also activated Th1 cells, but to a higher degree than that of Th2 cells, thereby causing a shift in the balance between Th1/Th2 cells towards a Th1 polarization. Indeed, IL-7 treatment caused a 7-fold increase of IFN-γ, 37 ± 13 compared to 253 ± 133 pg/mL, p < 0.05 (figure 6B). To measure the effect of IL-7 on the Th1/Th2 balance the ratio between IL-4/IFN-γ^-3 ^was computed; this ratio decreased significantly in allergen-challenged CD4^+ ^cells that were treated with IL-7 compared to untreated cells, 30 compared to 15, p < 0.05 (figure 6C). Thus, by a relatively higher Th1 than Th2 stimulation IL-7 signaling had a Th1 polarizing effect, which is consistent with an inhibitory role of IL-7R in allergic inflammation.

## Discussion

Identification of novel genes in high-throughput studies of complex diseases may require choosing between hundreds of genes. Since disease genes tend to interact with each other [1,2] the choice may be facilitated by searching for modules of disease genes that are co-expressed and interact [3,4].

It is not known if the module-based analytical strategy can also be applied to complex diseases other than cancer, which result from altered interactions between multiple cells and tissues. The hypothesis in this study was that we could find and validate a disease module in allergic disease and use this to find a novel disease gene.

In a recent study of breast cancer four known disease genes were used as references to construct a module of disease genes [3]. However, since allergy involves many different cells and tissues such an approach would result in a large number of reference genes to prioritize between. We therefore first performed an integrated analysis of gene expression microarray data from different forms of allergic diseases to find a common pathway. That pathway was used to find a reference gene for module construction. T cell receptor (TCR) signaling was the pathway with the highest statistical score. While many of the genes in the TCR pathway had not been previously described in human allergic disease, this pathway is in accordance with the current understanding that T cells play a key regulatory role in the disease [19]. On the other hand, it is important to emphasize the limitations of the study, for example that important low-abundance genes such as many cytokines are not detectable by gene expression microarray analyses. Only a small proportion of the different cells and tissues involved in allergic diseases were examined. Also, pathways other than TCR signaling may be important for the disease. Finally, the identification of pathways is restricted by current knowledge of gene functions and interactions.

We proceeded to study the TCR pathway genes in CD4^+ ^cells from patients with seasonal allergic rhinitis (SAR). This lead to the identification of six differentially expressed genes, all of which could potentially be used as putative reference genes. However, in order to select one we focused on *ITK*, because of its involvement in the polarization of Th2 cells and its location in a chromosomal susceptibility region for allergy [18]. The relevance of *ITK *was validated in a mouse model of allergy. The number inflammatory cells in both the upper and lower airways was significantly decreased in allergen-challenged *ITK-/- *compared to wild type mice. Next, we identified and validated a transcription factor (TF) that was shared by *ITK *and two other TCR pathway genes. This TF, *GATA3*, was used to construct a hypothetical module based on known gene interactions and co-expression data. This module contained a set of genes involved not only in TCR signaling, but also Th2 cell differentiation and homing. However, rather than being organized in distinct pathways these genes were highly interconnected. This suggests that complex cellular behavior like Th2 cell homing and activation is based on the co-regulation of genes from different pathways by common TF such as GATA3.

Validation studies showed that the module could be replicated in an independent material. Moreover, since corticosteroids are effective in treating allergic disease we tested if this treatment would also *reverse *the expression levels of the genes that had changed after allergen-challenge. Indeed, this was true for a significant proportion of the module genes. Taken together these findings lent strong support for our hypothesis that a relevant module could be found and validated in allergic disease.

We also hypothesized that the module could be used to identify a novel disease gene. Indeed, eleven of the 23 module genes had not been previously described in allergy, but may have important roles. For example, *SH2D1A *that promotes Th2 activation [20] increased in expression following allergen-challenge. However, while there are several options for functional studies of individual genes, it is less clear how to select one for such studies. Recent work indicates that genes with many interactions are functionally more important than those with few interactions [1,2]. We therefore reasoned that since disease genes tend to interact the novel gene that had most interactions with known disease genes would be most likely to be pathogenic. Analysis of the connectivity of the novel genes led to the identification of *IL7R*, whose relevance was supported by converging observations; *IL7R'*s interactions included two signature genes for Th1 and Th2 activation, namely *IFNG *and *IL4R *[21]; the expression levels of *IL7R *showed highly significant decreases in the allergen-challenged CD4^+ ^cells which were reversed by treatment with corticosteroids. Such expression changes are characteristic for genes that inhibit allergic inflammation [22,23]. These observations led to the hypothesis that *IL7R *inhibited Th2 cell activation. To test this hypothesis we treated allergen-challenged cells with IL-7. However, this resulted in increased rather than decreased IL-4 production, indicating that IL-7 activated Th2 cells. We therefore hypothesized that IL-7 stimulation would result in a relatively stronger activation of Th1 than Th2 cells. Indeed, IL-7 did cause an increased production of the Th1 cytokine IFN-γ, which significantly decreased the ratio between IL-4/IFN-γ. Thus, by inducing a relatively higher Th1 than Th2 activation, IL-7 signaling has a Th1 like effect, which is consistent with an inhibitory role of IL-7R in allergic inflammation. This has not been previously described, but increased expression of *IL7R *has been shown in Th1-like disorders like multiple sclerosis [24]. This suggests that this gene may have therapeutic potential in inflammatory diseases that depend on an altered balance between Th1 and Th2 cells.

The identification of *IL7R *in this study also supports the second hypothesis, namely that a module-based approach can be applied to find novel genes. It is also of note that although there is limited information about gene interactions, analyzing the number of interactions with known disease genes is a useful complementary method to prioritize between novel module genes for validation studies.

## Conclusion

The identification of novel genes by high-throughput studies of complex diseases is complicated by the large number of potential genes. Since disease genes tend to interact, one solution is to arrange them in modules based on co-expression data and known gene interactions. In this study we validated the hypothesis that such a module could be a) found and validated in allergic disease and b) used to find and validate one ore more novel disease genes. We propose that the analytical principles may be generally applicable to complex diseases.

## Methods

### Subjects analyzed with DNA microarrays

For the integrated analysis a total of 36 patient samples were analyzed with 40 DNA microarrays. A total of 27 control samples were analyzed with 31 DNA microarrays. These samples were used for steps 1–3 in the results. In addition, for step 4, coexpression studies were performed in T helper cells from 73 atopic patients and 19 non-atopic controls. These materials have been described in detail elsewhere [7-9,25]. The validation studies of hypothetical module genes in steps 5–7 were performed in CD4^+ ^cells from 19 patients, a) with and b) without allergen-challenge as well as c) with challenge and treatment with cortisone. Samples from each individual were analyzed with one microarray, unless otherwise stated. All gene expression microarray data, except those from the asthmatic patients, are derived from studies performed by the authors of this study. The materials can be summarized as follows:

#### Seasonal allergic rhinitis (SAR)

1) CD4^+ ^T cells from 22 patients with SAR were examined before and after stimulation with grass pollen extract. CD4^+ ^T cells will subsequently referred to as T helper cells. The cells were obtained outside of the pollen season. 2) Nasal fluid cells from 18 patients before the pollen season sampled before allergen challenge as well as one and six hours after allergen challenge. In addition nasal fluid cells were obtained from symptomatic patients during season. The nasal fluid cells from each time point were pooled and analyzed with one DNA microarray GeneChip at each time point. 3) Skin biopsies from ten patients sampled one hour after subcutaneous allergen challenge 4) Nasal polyps from two patients with SAR analyzed before and after treatment with glucocorticoids. Each polyp was analyzed with duplicate microarrays on each occasion.

#### Asthma

T helper cells from seven patients with mild asthma and five patients with severe asthma.

#### Eczema

Skin biopsies from ten patients with atopic eczema who fulfilled the criteria of Williams.

#### Controls

1) T helper cells from four healthy controls. 2) Nasal fluid cells from twenty healthy controls. The nasal fluid cells were pooled and analyzed with one DNA microarray GeneChip. 3) Nasal polyps from two patients without allergy, before and after treatment with glucocorticoids. Each sample was analyzed with duplicate DNA microarrays. 4) Skin biopsies from ten healthy controls. 5) Allergen-challenged skin biopsies from ten healthy controls (these individuals were not the same as in study 4). The raw array data are available at Gene Expression Omnibus , pending processing, accession numbers GSE6012, GDS266 and GSE473.

### Allergen challenge of peripheral blood mononuclear cells

Peripheral blood mononuclear cells were prepared and stimulated with grass pollen extract with or without hydrocortisone as described [7]. For gene expression studies, T helper cells were enriched from the allergen-challenged PBMC using anti-CD4-coated paramagnetic microbeads and a MACS (magnetic cell sorter) system according to the instructions of the manufacturer (Miltenyi Biotec GmbH, Bergisch Gladbach, Germany). cRNA was extracted from 200 ng total RNA using Ambion's Illumina RNA TotalPrep Amplification kit (Ambion, Inc., U.S.A.). In vitro transcription (IVT) reaction and cRNA biotinylation was performed overnight (14 h). The RNA/cRNA concentrations where checked using Nanodrop ND-1000 before and after the amplifications. cRNA quality was controlled by BioRad's Experion electrophoresis station (Bio-Rad Laboratories, Inc., CA, U.S.A.).

### Microarray hybridization and data analysis

All gene expression profiling studies except the validation data in allergen-challenged CD4^+ ^cells were performed using DNA microarrays measuring the expression of 22,283 genes and variants (HuGe U133A GeneChip, Affymetrix, Santa Clara, CA) as previously described [2]. The validation studies of allergen-challenged CD4^+ ^cells from patients with SAR were performed DNA microarrays measuring the expression of 47293 genes (Illumina, San Diego, CA). The analyses were performed according to manufacturer's instructions and the MIAME guidelines. Identification of disease-associated genes was performed as described in the additional file 1.

### Network-based analysis of disease-associated genes

The Ingenuity Pathways Analysis application (IPA) was used to organize the disease-associated genes into networks of interaction and to find modules of functionally related genes that correspond to pathways as previously described [7]. A detailed description is given in the additional file 1.

### Identification of a shared framework of transcription factor binding sites for the T cell receptor pathway

A shared framework of transcription factor binding sites (TFBS) for genes of the T cell receptor pathway was identified using a set of *in silico *genomics tools (see additional file 1).

### Animal Experiments

An exploration of *ITK*-deficient mice in a validated mouse model of allergic airway inflammation was used to assess the involvement of ITK in manifesting characteristic pathogenic features of an allergic inflammation. Tissue eosinophilia was selected as key parameter since this feature is strongly associated with allergy. Furthermore, the development of an airway eosinophilia in the present model has been demonstrated to be strictly dependent on a functional Th2 response [26] (see additional file 1).

### Analysis of the effect of IL-7 on Th1/Th2 polarization

PBMCs from 5 patients with seasonal allergic rhinitis were purified as described above. 2.5 × 10^6 ^cells in 2 mL culture medium (cells/mL) were stimulated with 100 μg/mL grass or pollen extract in the presence or absence of 10 ng/mL rhIL-7 (R&D systems Ltd, UK). The culture supernatants were collected after 7 days. IL-5, IL-13 and IFNγ cytokines were analyzed in the supernatants using human interleukin Quantikin ELISA kits from R&D systems according to the manufacturer's protocol. IL-4 Quantikin HS kit was used to for quantitative determination of human IL-4 concentrations in cell culture supernatants.

### Statistical analysis

Paired comparisons were performed using the Wilcoxon signed-rank test, while unpaired ones were performed using the Mann-Whitney U-test. The Spearman rank correlation test was used to study correlations.

## Abbreviations

SAR: Seasonal Allergic Rhinitis; SNP: Single Nucleotide Polymorphism; TCR: T Cell Receptor; Th2: T helper cell type 2; TF: Transcription Factor; TFBS: Transcription Factor Binding Site.

## Authors' contributions

RM carried out the molecular studies and drafted the manuscript. BA, MHZ and JE carried out the immunological analyses. ML and AP performed the computational analyses. LOC participated in the design of the study. MB conceived of the study, and participated in its design and coordination and helped to draft the manuscript. All authors read and approved the final manuscript.

## Supplementary Material

Additional file 1 **The document describes additional methods, analysis data of IL-5 and IL-13 in allergen-challenged CD4^+ ^cells with and without IL-7 and additional figure 1 which describes the Pathway analysis of disease-associated genes.** Click here for file
