# Supplementary material for: A module-based analytical strategy to identify novel disease-associated genes shows an inhibitory role for interleukin 7 Receptor in allergic inflammation
Source: BMC Syst Biol. 2009 Feb 12;3:19. doi: 10.1186/1752-0509-3-19 (PMC2653464; doi:10.1186/1752-0509-3-19)
Supplement: Additional file 1 — The document describes additional methods, analysis data of IL-5 and IL-13 in allergen-challenged CD4+ cells with and without IL-7 and additional figure 1 which describes the Pathway analysis of disease-associated genes. [file 1752-0509-3-19-S1.doc]

### Additional FILE 1

### Methods

### Microarray Data Analysis

Control and patient data were handled separately. For each, the Affymetrix Micorarray Suite MAS5.0 was used to convert raw scanned images into intensity calls. Probe set values were averaged, log transformed and z-scored. Genes were eliminated from consideration if, under all conditions, they were either weakly expressed or their expression levels did not significantly change. Pearson’s correlation coefficients were computed and used to populate symmetric correlation matrices with values in the range [-1, 1]. Negative and positive correlations are equally meaningful when measuring putative co-regulation, and so the absolute value of each correlation coefficient was taken. Thresholds were then used to transform matrices into correlation graphs. Vertices were joined by an edge if and only if the corresponding genes were correlated at or above the appropriate threshold. For each threshold considered, maximum clique computations were performed using codes synthesized from vertex cover duality and the theory of fixed parameter tractability [1]. For quality monitoring, Affymetrix control probe sets were retained. Their frequency was found to be in accordance with chance. To capture biological significance and balance edge densities, a threshold of .92 was selected for control data while .90 was chosen for patient data. Maximal cliques were extracted using the methodology and prioritized by gene ontology. Only genes that passed three differential analysis screens were selected for detailed scrutiny. First, individual transcripts had to be “differentially expressed,” a well-known measure of significance. Next, pairs of transcripts had to be “differentially correlated” [2], which we define here as correlated at 0.85 or above in one treatment and at 0.25 or below in the other. Finally, genes had to be “differentially cliquified” [3] which we define in its simplest form as highly represented in the clique lists of one treatment and poorly represented in the other.

**Network-based analysis of gene expression microarray data**

The Ingenuity Pathway Analysis tool (IPA) was used to organize differentially expressed genes into networks of interacting genes and to find modules of functionally related genes that correspond to pathways. The IPAconsists of a global network that is based on reviewing and extracting mammalian gene interactions from review of more than 200,000 articles in 275 peer reviewed journals. The findings for the IPA were manually curated and assigned to ontologic classes by content and modeling experts. Interactions were also inferred algorithmically by combining functional and structural information about proteins. Every interaction can be referenced back to a MedLine ID within the application. Identification of networks formed by the differentially expressed genes was done in a stepwise manner:

1. Differentially expressed genes are scored on the basis of the number of specific interactions found within the IPA global network. Focus genes were identified as those having direct or indirect interactions with other genes in the IPA database.

2. The specificity of connections for each focus gene was calculated by the percentage of its connections to other differentially expressed genes. A network was then constructed, starting with the gene with the highest specificity of connections. Each network contained a maximum of 35 genes. This process was repeated for the remaining genes to construct new networks. As the number of focus genes is reduced as a result of their inclusion in previous networks, genes from the global network not in the original list but with significant specific connectivity to the growing network can be included into those new networks.

3. A statistical algorithm based on the Fischer exact test was used to rank the networks. Identification of pathways was based on using canonical pathways in the IPA as templates for the differentially expressed genes. Again using the Fischer exact test, the resulting pathways were ranked according to their correlation with the template in the IPA.

### Identification of a shared framework of transcription factor binding sites for the T cell receptor pathway

A shared framework of transcription factor binding sites (TFBSs) for genes of the T cell receptor pathway was identified using a set of *in silico* genomics tools, i.e. Gene2Promoter, DiAlign, FrameWorker, and ModelInspector (Genomatix, Munich, Germany). Evolutionarily conserved frameworks containing 2 TFBSs were identified and searched to isolate a TCR pathway gene of importance for allergic inflammation. Based on experimental studies, *ITK* was chosen as such a gene. The initial set of conserved frameworks was subsequently used as a seed to find and refine matching frameworks among the other pathway genes. The initial, conserved frameworks were identified in proximal promoter regions that were defined as 500 nt upstream of and 100 nt downstream from the transcription start site (TSS). The sequences were retrieved using Gene2Promoter from three species (*H. sapiens* , *M. musculus* , and *R. norvegicus* ). Frameworks of different combinations of 2 TFBS conserved in TFBS distance and orientation were identified using FrameWorker. The default settings for position weight matrices were used. The conserved frameworks were then compared with the promoter regions of all genes of the TCR pathway to find a subset of pathway genes that shared frameworks using ModelInspector. Promoters from genes sharing frameworks were then extracted by Gene2promoter and subjected to FrameWorker to identify a generalized framework for this subset. The relevance of this framework was tested by examining the expression levels of the corresponding TF in allergen-challenged T lymphocytes that had been analyzed by DNA microarrays as described above.

**Animal experiments**

Briefly, wild type mice (C57/Bl6) and age-matched mice that carry targeted and *ITK*^-/-^ alleles were actively immunized against ovalbumin (OVA, grade III, Sigma, St Louis, MO) at day 1 by an intraperitoneal injection (7.5 μg OVA + 1.5 mg AlOH_3_). At 21-28 days after i.p., immunization animals were challenged daily with aerosolized allergen (30 minutes 1% OVA in saline). At 24 h after the last challenge and nasal and lung tissues were collected for histological analysis. Eosinophils were detected in cryo sections by histochemical staining of cyanide-resistant eosinophil peroxidase (EPO) and Normasky optics. Eosinophils were counted around the bronchi and large pulmonary vessels in a circular tissue region spanning from the epithelial/endothelial basement membrane to a tissue depth of 250 μm. Periodic Acid Schiff (PAS) staining of mucin granules was used to study the numbers and distribution of airway goblet cells. Briefly, the number of PAS-positive cells were counted along the bronchial epithelium and correlated to the length of the epithelial lining (which was determined using digital image analysis). All quantifications were performed in a blinded manner. The local ethics committee, Lund/Malmö, Sweden approved all animal procedures.

**Results**

### Analysis of IL-5 and IL-13 in allergen-challenged CD4^+^ cells with and without IL-7 stimulation

The IL-5 level in allergen-challenged CD4^+^ cells (mean ± SEM) was 117 ± 47 compared to cells stimulated with allergen and IL-7, 326 ± 85 pg/mL, p<0.05. The corresponding figures for IL-13 were 134 ± 41 compared to 582 ±1 44 pg/mL (p<0.05).

**References**

1. Downey RG, Fellows MR: *Parameterized Complexity*. New York: Springer; 1999.

2. Voy BH, Scharff JA, Perkins AD, Saxton AM, Borate B, Chesler EJ, Branstetter LK, Langston MA: **Extracting gene networks for low-dose radiation using graph theoretical algorithms**. *PLoS computational biology* 2006, **2**(7):e89.

3. Lansgton MA, Perkins AD, Saxton AM, Scharff JA, Voy BH: **Innovative Computational Methods for Transcriptomic Data Analysis Proceedings**. In *ACM Symposium on Applied Computing: 2006; Dijon, France*; 2006.

**Figure 1**

**
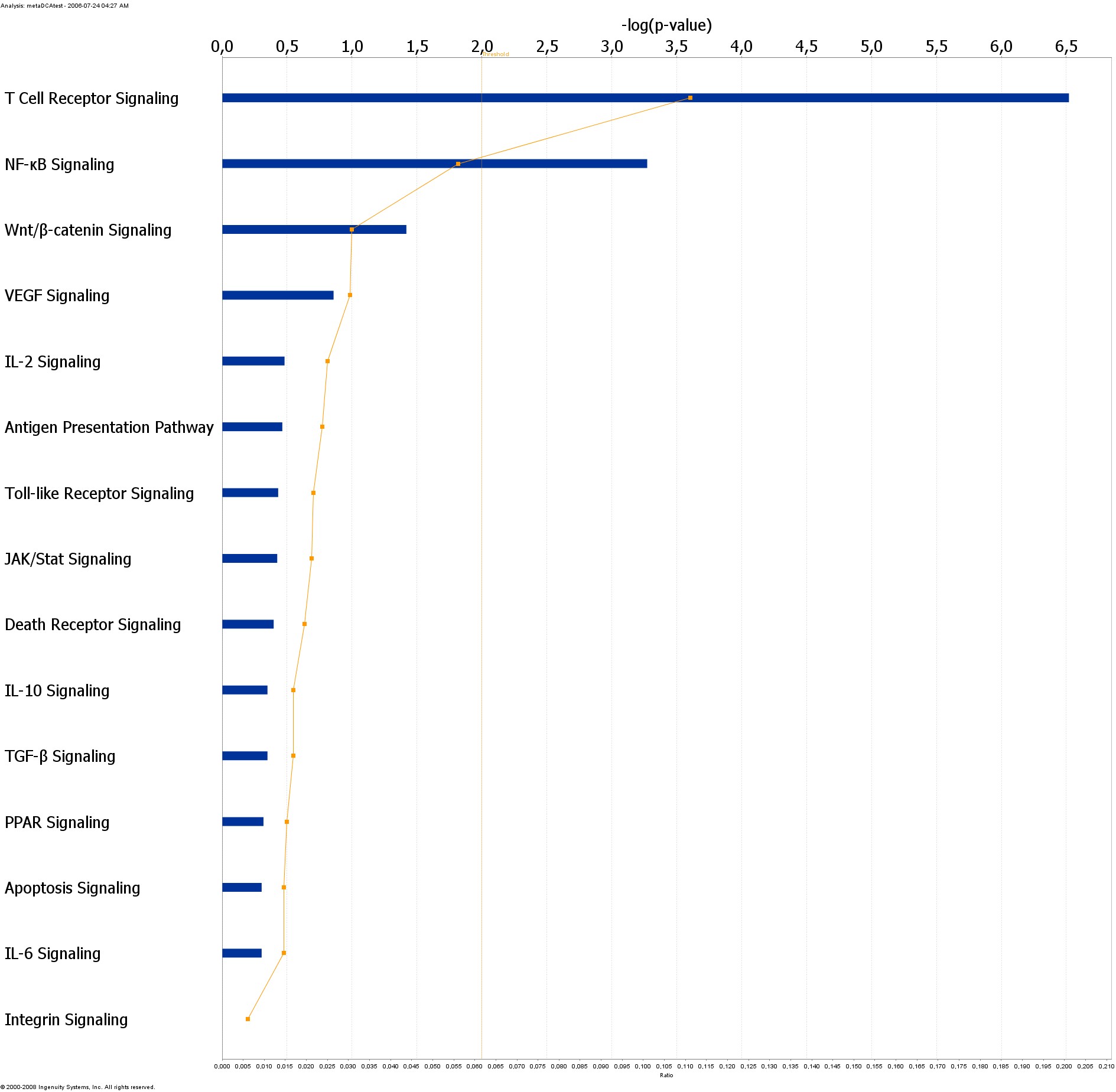
**

### Legend to figure

**SI Figure 1.** Pathway analysis of disease-associated genes. The Fischer’s exact test was used to rank the pathways statistically. The yellow line indicates the significance threshold of p<0.01.
